# Supplementary material for: Anthocyanin Accumulation Provides Protection against High Light Stress While Reducing Photosynthesis in Apple Leaves
Source: Int J Mol Sci. 2022 Oct 20;23(20):12616. doi: 10.3390/ijms232012616 (PMC9604341; doi:10.3390/ijms232012616)
Supplement: Supplementary file 1 [file ijms-23-12616-s001.zip › ijms-1975915-Supplemental figures.pdf]

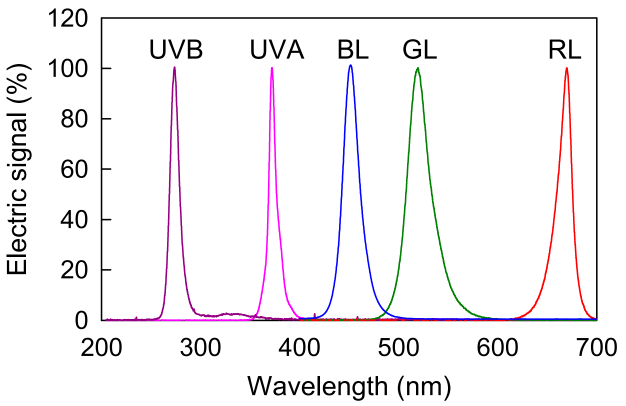

Figure S1 The spectra of different light sources used in this study.

BL, blue light; GL, green light; RL, red light.

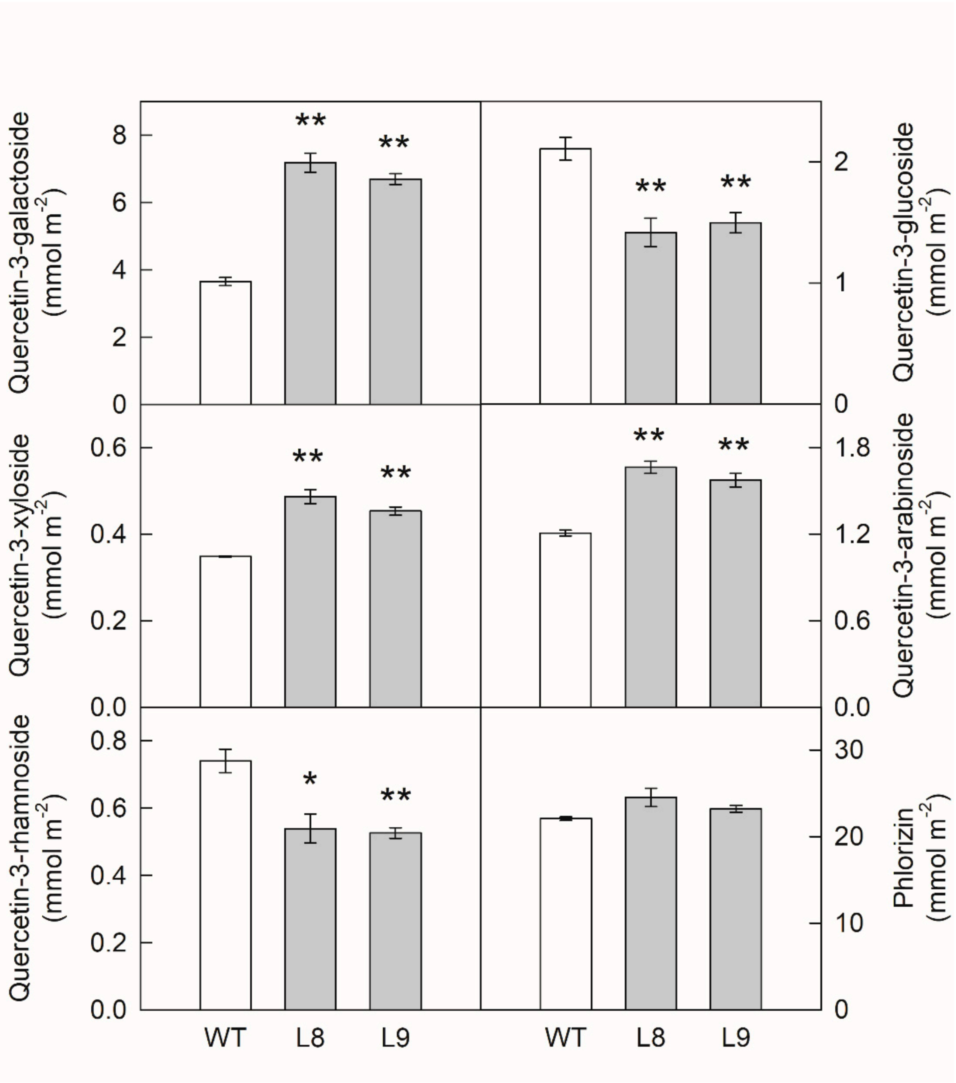

Figure S2 The contents of flavonol compounds and phlorizin in leaves of 'Galaxy Gala' (WT) and its transgenic lines (L8, L9) with *MdMYB10* overexpression.

Data are means ± SE (n = 5). The "*" or "**" indicates a significant difference at $P < 0.05$ or $P < 0.01$, respectively, *t*-test.

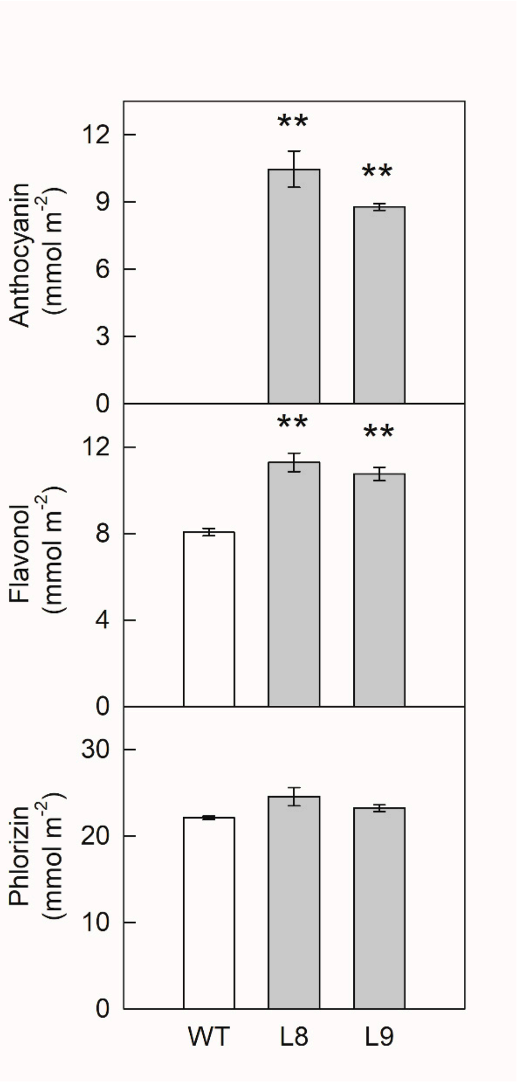

Figure S3 Flavonoid content in the bark of young branches of 'Galaxy Gala' (WT) and its transgenic lines (L8, L9) with *MdMYB10* overexpression.

Data are means ± SE (n = 5). For the statistical analysis, "**" indicates a significant difference at $P < 0.01$, *t*-test.

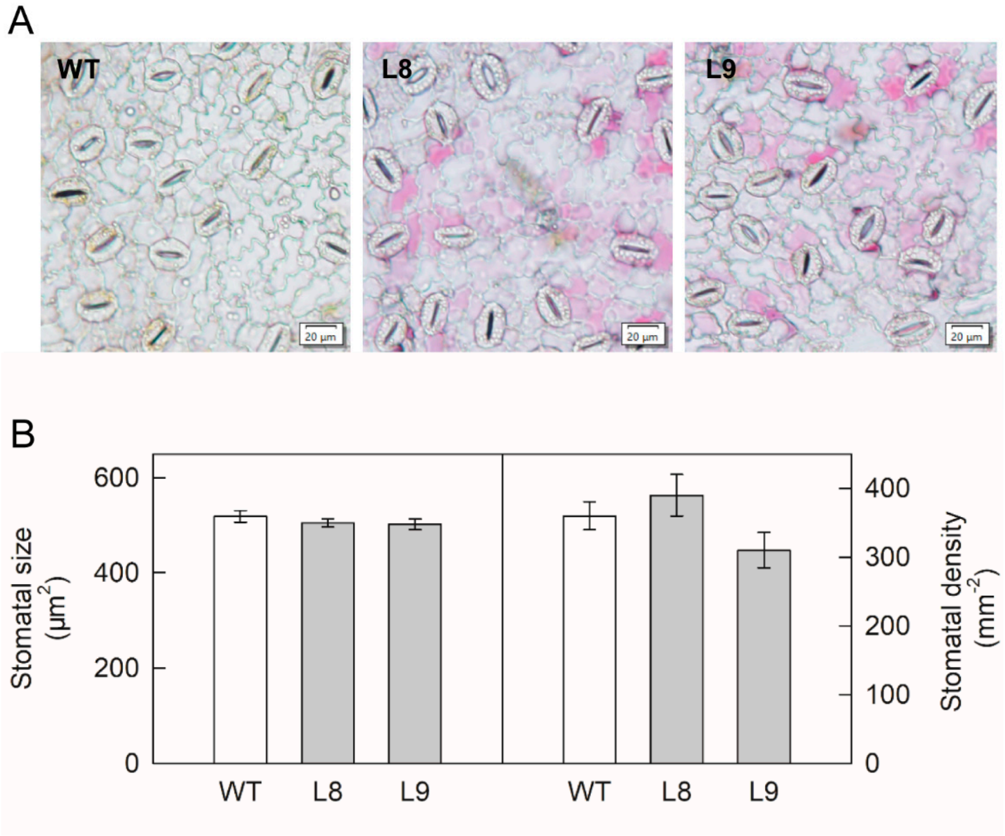

Figure S4 Stomatal phenotypes (A), size, and density (B) in leaves of 'Galaxy Gala' (WT) and its transgenic lines (L8, L9) with overexpression of *MdMYB10*. Five biological replicates were performed for each point.

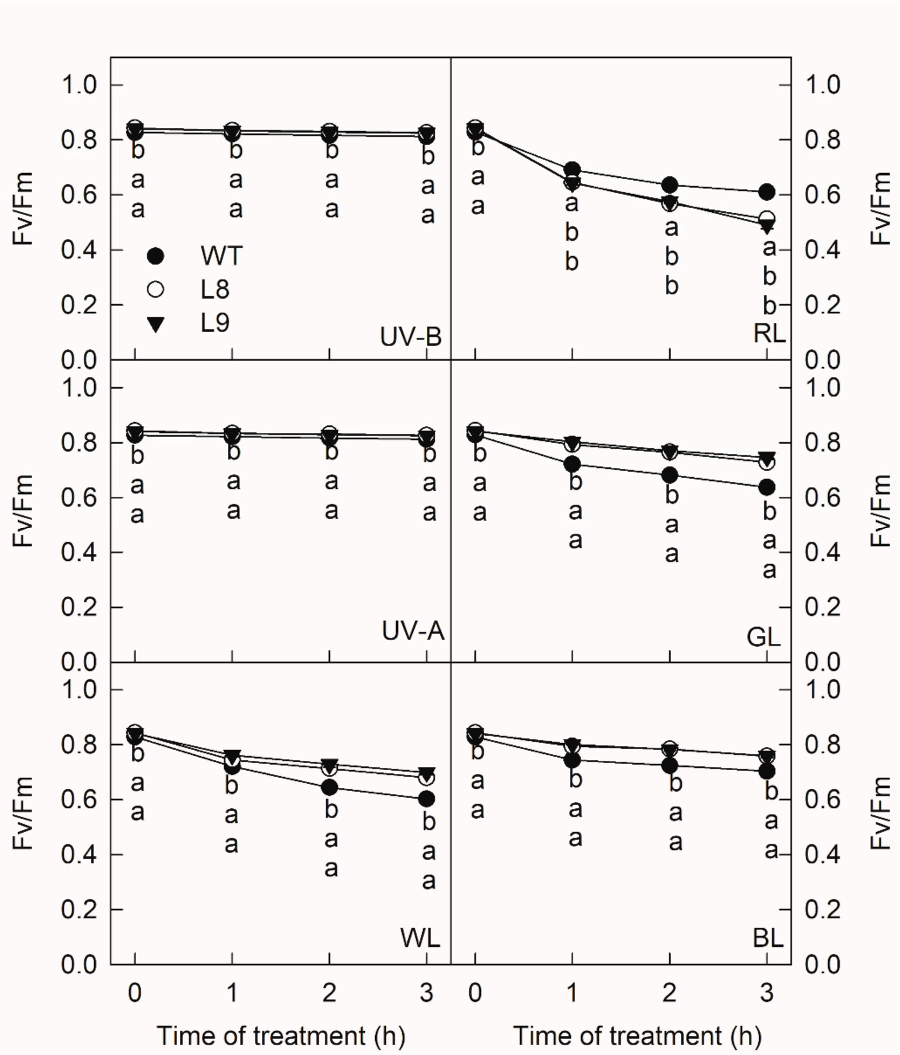

Figure S5 Relative changes in the maximum quantum yield of PSII (Fv/Fm) in leaves of 'Galaxy Gala' (WT) and its transgenic lines (L8, L9) with *MdMYB10* overexpression after different treatments under laboratory conditions.

WL, white light, RL, red light; GL, green light; BL, blue light. Data are means ± SE (n = 5). Different small letters below the symbols indicate a significant difference among genotypes at P < 0.01, LSD.
